# Immersive Virtual Reality for Pediatric Pain

**DOI:** 10.3390/children4070052

**Published:** 2017-06-23

**Authors:** Andrea Stevenson Won, Jakki Bailey, Jeremy Bailenson, Christine Tataru, Isabel A. Yoon, Brenda Golianu

**Affiliations:** 1 Department of Communication, Cornell University, 417 Mann Library Building, Ithaca, NY 14853, USA; a.s.won@cornell.edu; 2 Department of Anesthesiology and Perioperative Medicine, Stanford University, 300 Pasteur Dr. H3580A, Stanford, CA 94305, USA; jakki7@stanford.edu (J.B.); bailenso@stanford.edu (J.B.); ctataru5@stanford.edu (C.T.); iayoon@stanford.edu (I.A.Y.)

**Keywords:** Virtual reality, pediatric pain, procedural pain, nonpharmacological, rehabilitation

## Abstract

Children must often endure painful procedures as part of their treatment for various medical conditions. Those with chronic pain endure frequent or constant discomfort in their daily lives, sometimes severely limiting their physical capacities. With the advent of affordable consumer-grade equipment, clinicians have access to a promising and engaging intervention for pediatric pain, both acute and chronic. In addition to providing relief from acute and procedural pain, virtual reality (VR) may also help to provide a corrective psychological and physiological environment to facilitate rehabilitation for pediatric patients suffering from chronic pain. The special qualities of VR such as presence, interactivity, customization, social interaction, and embodiment allow it to be accepted by children and adolescents and incorporated successfully into their existing medical therapies. However, the powerful and transformative nature of many VR experiences may also pose some risks and should be utilized with caution. In this paper, we review recent literature in pediatric virtual reality for procedural pain and anxiety, acute and chronic pain, and some rehabilitation applications. We also discuss the practical considerations of using VR in pediatric care, and offer specific suggestions and information for clinicians wishing to adopt these engaging therapies into their daily clinical practice.

## 1. Introduction

Children have always enjoyed games of “pretend.” While immersed in a game, they often become deeply absorbed and able to ignore aversive stimuli. Immersive virtual reality (VR) is a promising and engaging intervention that may help to decrease pain and anxiety for children undergoing painful procedures and suffering from acute pain. In the context of their medical care, children may also endure chronic pain and discomfort. Because VR makes it possible to transform how patients perceive their bodies, it allows other, novel interventions that are possible in no other medium. Beyond providing distraction and enjoyment, virtual reality may provide a corrective psychological and physiological environment, and can facilitate rehabilitation for pediatric patients suffering from chronic pain, as well as neurorehabilitation for children suffering from stroke and cerebral palsy. With the advent of inexpensive consumer VR systems, the opportunities to research and deploy VR in the clinic have expanded. However, the powerful and transformative nature of many VR experiences may also pose some risks and should be utilized with caution in developing therapeutic VR interventions [1].

In this paper, we will review recent literature in pediatric virtual reality for procedural pain and anxiety, acute and chronic pain, and some rehabilitation applications. We will also discuss clinically relevant characteristics of VR experiences, such as the aspects of presence, interactivity, customization, social interaction, and embodiment. We will review how each of these requires special consideration, and in some cases adaptation of the hardware or software, for a child population. Finally, we will discuss the practical considerations of some currently available consumer VR systems, and offer specific suggestions and information for clinicians wishing to adopt these engaging therapies.

## 2. Review of Virtual Reality in Pediatrics

We conducted a review evaluating articles that describe the use of VR in pediatric procedural, acute and chronic pain. (see Table 1) We included articles detailing case studies or randomized trials of the use of VR. Where such studies were not available, we referred to adult studies that may offer insights into the potential use in pediatric populations, which are specifically described as pertaining to adult populations. We will discuss the selected articles and themes as they are relevant to the improved care of pediatric patients through VR. These articles can be roughly divided into two main areas of clinical relevance: acute and procedural pain and anxiety, and chronic pain and neurorehabilitation.

### 2.1. Acute and Procedural Pain

Acute pain is pain directly related to a temporary injury and typically lasts a short period of time (<6 weeks). Procedural pain and anxiety refers to distress derived from medical procedures, including intravenous (IV) injections, vaccinations, anesthesia administration, and other needle-related procedures, as well as other procedures required as part of routine care, such as burn wound dressing changes.

Virtual reality was first used to manage acute pain during painful repetitive dressing changes in patients with burn wounds [2]. Schmitt et al. demonstrated a reduction in pain of 27–44% in pediatric patients who participated in the virtual environment featuring a wintery scenario, called “SnowWorld”, while undergoing dressing changes [3,4]. Jeffs et al. corroborated the efficacy of VR in burn wound pain management in adolescents [5]. In a randomized three-armed trial, 30 adolescents were assigned to standard care, watching a movie, and VR engagement with the “SnowWorld” virtual environment. The VR treatment group reported a decrease in pain compared to the passive distraction (movie) group, (difference 23.7 points decrease in pain score out of 100 total), 95% confidence interval (CI): 2.4–45.0, *p* = 0.029). In comparison to standard care, the VR group showed decreased pain as well, though the difference was not statistically significant. Of note, the VR group was the only one in which the patients’ pain during the actual dressing change procedure was reported to be less than the pre-procedure pain [5].

Brown et al. performed a randomized controlled trial of an interactive VR intervention entitled “Ditto” and showed not only a decrease in self-reported pain and anxiety in pediatric patients undergoing dressing changes, but also an increased rate of epithelialization and faster wound healing in those patients undergoing VR intervention for their procedures (−2.14 days (wounds healed 2.14 days faster), CI: −4.38 to 0.1, *p* = 0.061), which was significantly faster when adjusted for mean burn depth (−2.26 days, CI −4.48 to −0.04, *p* = 0.046) [6]. Lastly, Miller et al. showed that not only was pain reduced during dressing changes, but when a multimodal intervention was utilized, the procedure required less time to complete (*p* < 0.05) [7]. Additional notable studies investigating the efficacy of VR distraction on pain and anxiety in children and adults undergoing severe burn wound care are referenced below [8,9].

Virtual reality has also proven successful at lessening procedural pain and distress related to IV placement and other needle-related procedures. In a 2006 study, Gold et al. performed a randomized control trial on 20 pediatric patients requiring IV placement [10]. The VR group received a multisensory VR experience including visual stimulation from a HMD device, tactile feedback, and music, for 5 min prior to IV placement until 5 min after placement. The control group received local anesthetic spray, but no VR intervention for the procedure. They were permitted to utilize the VR equipment for 3 min following IV placement. While children in the control group experienced a four-fold increase in pain as measured by the Wong-Baker FACES scale [11], the VR group showed no increase in affective pain following IV placement [10]. Nilsson et al. performed a similar study (*N* = 42) using a screen-based three-dimensional (3D) program [12]. Pediatric and adolescent patients undergoing venous punctures or subcutaneous venous port device insertion were randomized either to this non-immersive VR and standard care, or standard care alone. Patients did not report a significant decrease in self-reported pain for either condition; however, they did report that the VR distraction was a pleasant experience that succeeded at distraction. (We note that this intervention was not immersive).

Beyond VR’s use in acute pain, it can potentially be used to make patients more familiar and comfortable with procedures. Most hospital procedures are accompanied by some stress or anxiety on the part of pediatric patients. He et al. looked at the effect of therapeutic play on perioperative anxiety, negative emotional manifestation, and postoperative pain [13]. Ninety-five children were assigned randomly to receive one hour of face-to-face therapeutic play involving objects to be used in the operation, or no intervention. Those children that participated in therapeutic play exhibited significantly lower levels of anxiety and negative emotion manifestation associated with their upcoming operation, and even showed lower levels of post-operative pain [13]. Virtual reality has the potential to familiarize children with the operation environment in a safe, controlled and playful way, and potentially decrease their pain and anxiety.

### 2.2. Chronic Pain

Unlike procedural or acute pain, chronic pain is persistent for a period greater than six weeks, often for months or years. It may include chronic headache, abdominal, limb, joint or back pain, neuropathic or sympathetically maintained pain such as complex regional pain syndrome (CRPS), and pain related to other medical conditions. In addition to symptom control, interventions often strive to maintain or improve function and minimize disability. While there is little research on using VR for the treatment of chronic pain in children [14], promising results in adults indicate that further research in this area is advisable. Below, we describe the use of virtual reality in neuromodulation, in physical therapy, and in biofeedback. We describe the limited research in children, and attempt to extend relevant research in adults to pediatric patients.

Virtual reality may have the potential to amplify the neuromodulatory effects of traditional mirror visual feedback (MVF), which can modify patients’ pathological cortical representations in the group of chronic pain syndromes that includes phantom limb pain, complex regional pain syndrome and fibromyalgia. Mirror visual feedback was first applied to adult patients suffering from phantom limb pain (PLP) by Ramachandran and Rogers-Ramachandran [15]. This technique may exert some of its benefits by encouraging the remapping of motor and somatosensory cortices, allowing a return to a homeostatic processing mechanism and facilitating the improvement or resolution of the painful experience. Mirror visual feedback was also found to be of benefit for CRPS in a randomized controlled trial with adult patients [16]. Virtual reality has also been used in an open-label case study of five adults with CRPS, four of whom reported reduced pain intensity over five to eight sessions [17].

Besides providing a new medium for mirror therapy, VR also allows other avatar configurations to be altered. For example, patients can also experience augmented motion such that a small motion in the physical world maps to a large motion in the virtual one, or vice versa. Thus, a small movement of the arm made in real life could be rewarded by a larger, more apparent arm movement in VR. Conversely, for patients prone to guarding, movements made by patients in real life could be depicted as more restrained in the virtual world [18], which might encourage such patients to move more freely. Two studies demonstrated that adult patients with neck pain moved further and reported less pain as a result of movement when VR was used to reduce their apparent movement [18,19]. In the pediatric population, there is currently one feasibility study exploring the neuromodulatory effects of VR. Won et al. showed that a VR experience with MVF and movement augmentation properties was well tolerated by pediatric patients with CRPS during physical therapy sessions [20].

The hypothesis supporting the efficacy of neuromodulatory chronic pain therapies is founded in the association between brain connectivity changes and improvement in chronic pain symptoms. A study conducted by Lebel and colleagues found significant changes in the somatosensory processing of symptomatic pediatric CRPS when compared to post-treatment CRPS patients and control patients [21]. Because this processing resumes normality after months of traditional physical therapy, it is possible that directly altering somatosensory processing (i.e., via mirror therapy), might itself be helpful in resolving pain. Becerra et al. and Simons et al. [22,23] both showed changes in connectivity in various intrinsic brain networks after therapies that reduced pain and improved function. It is still under debate whether the association of successful recovery from CRPS symptoms is directly related to changes in brain connectivity or brain plasticity, and further research is required to determine what other factors may be involved in remapping somatosensory processing [24]. However, another piece of evidence supporting this hypothesis comes from graded motor imagery, a therapy that involves the imaginary mental rotation of one’s limbs. Adult studies have shown that this therapy is associated with both changes in connectivity in the central nervous system as well as reduction in pain [25], and pediatric patients may also benefit from such therapy.

As discussed above, VR offers an engaging opportunity for children to practice motions that would be impossible or unsafe in the real world [26]. Especially in diseases like cerebral palsy, where motion is difficult, the opportunity to engage in active/repetitive motor and sensory practice can increase neuroplasticity and allow for learning to overcome some limitations of the disease [27]. In a study by Biffi et al., 12 children with Acquired Brain Injury (ABI) participated in 10 sessions using an interactive VR system called GRAIL (Gait Real-time Analysis Interactive Lab); treatment sessions led to improvement in walking abilities and enhanced engagement during training, suggesting that VR may play a role in the field of rehabilitation [28]. Although not discussing efficacy, Meyns et al. demonstrated the feasibility of using VR training to improve balance in children with cerebral palsy after lower limb surgery [29]. For a more in-depth review on neurorehabilitation applications of VR, please refer to the following excellent review by Wang et al. [26].

In addition to alterations in neuronal representation, patients with chronic pain often exhibit a debilitating fear of inducing more pain through movement. This fear not only seriously affects patients’ ability to function; it can also inhibit physical therapy efforts. Trost et al. discusses the use of VR interventions to provide graded exposure treatment for pain-related fear and disability in adults with chronic low back pain [30]. Collado-Mateo et al. also found that a combined program of physical exercise and non-immersive VR improved adult patients’ mobility, balance, and fear of falling in adult fibromyalgia patients [31]. Senkowski et al. cite significant promise in the use of VR-assisted therapy to aid in the decrease of fear-avoidance behavior and improvement in distorted body images [32]. Fear-avoidance is noted to be an issue in the pediatric population with chronic pain [33]; however, no VR interventions have yet been specifically designed to address this phenomenon.

### 2.3. Other Applications

Another possible application is the use of a virtual pain coach. A VR experience could reinforce therapeutic movements and exercises in an out-of-clinic environment. This is especially useful for long-distance patients or patients who are unable to be physically present for other reasons [34]. The variety of VR therapy options makes it an effective intervention to integrate into existing interdisciplinary programs, combining medical treatment, physical therapy and psychological interventions [35].

Finally, the effects of VR may potentially be augmented by other techniques such as biofeedback or hypnosis. Biofeedback is the process of providing the patient with information on his or her own neurological data such as heart rate variability, temperature, or muscle tension. In a recent pilot study, non-immersive VR mirror visual feedback was used successfully in combination with biofeedback to treat pediatric patients with chronic headaches [36]. Virtual reality was also used in conjunction with hypnosis in an adult case report [37].

## 3. Qualities of Virtual Reality

In the next section, we will discuss five qualities of VR that are of interest in considering clinical applications for children: presence, interactivity, social interactions, customization, and embodiment. We will then summarize current research on how these qualities may be useful for treating children, and note some potential areas of caution (see Table 2).

### 3.1. Presence

Presence is the subjective feeling that the user is experiencing the environment and interactions in the virtual world are [38]. It is often measured by self-report, has been linked to physiological processes such as changes in hear rate and skin resistance [39] and behavioral measures such as not responding to stimuli in the real world [40]. Feelings of presence can be evoked by even a simple VR system, such as a 360° video viewed through a smartphone in a cardboard housing. However, a recent meta-analysis indicates that features such as improved tracking, stereoscopy, and wide field of view can make VR experiences feel more real [1]. VR is particularly promising as a distractor for procedural and acute pain because of the deep sense of presence created by virtual worlds. The immersive features of virtual reality technology immerse the patient with rich sensory stimuli, creating a realistic experience and effectively directing attention away from adverse stimuli [41,42].

Age may influence how virtual environments are experienced. For example, during burn wound care treatment with VR, pediatric patients reported higher levels of presence compared to adults receiving the same treatment [43]. Experiencing virtual environments as extremely real has important implications for how children behave and understand the world after the VR treatment. Baumgartner et al. showed that children’s brains process virtual experiences differently than adults and young children may require different types of immersive experiences [44]. During early childhood (e.g., three to five-years-old) children’s sense of fantasy and reality is rapidly developing [45,46]. Virtual reality can create realistic environments that may seem extremely real, particularly to young children.

Given that children may be more vulnerable to believing virtual experiences as real, virtual environments may need to be carefully selected and contextualized so that children are able to interpret their memories of the experience as derived from media rather than real life. In addition, for some age groups, debriefing post-VR experience may be necessary.

### 3.2. Interactivity

Virtual reality therapies incorporating tracked body movements allow for greater interactivity. In a study examining cold pressor-induced pain in 40 children of age 5–13, greater interactivity led to greater pain tolerance [47]. Children with disorders requiring intensive physical therapy, such as those with CRPS [20] or cerebral palsy [48], may benefit especially from interactive VR scenarios. Such scenarios can encourage them to engage in physical therapy while simultaneously increasing their pain tolerance.

High levels of interactivity, while effective in reducing pain, can be accompanied by a sense of nausea and increased potential for collisions with objects in the real world. Clinicians using VR as therapy should be aware of these minor risks and take appropriate precautions for pediatric patients. The more immersed users are in a virtual environment, the more the sensory input from the real world is occluded, and active users risk painful collisions.

Even adults can be at risk of falling or colliding with objects; during set-up, consumer VR systems require users to define a safe “play space”, and displays are equipped with safety warnings and reminders to be careful when users approach the edge of this area. As children move around a virtual space, they may come dangerously close to objects in the real world. Even if children are seated, they should be monitored. Careful spotting is necessary to prevent them from injuring themselves or bystanders by accidentally colliding with real-world objects.

In addition, although both children and adults can be prone to “cybersickness” or feeling nausea or dizziness, children may be less able to anticipate and articulate their discomfort. Caretakers and clinicians will need to be aware of the possible signs of sickness and develop ways to measure children’s discomfort in non-verbal and unobtrusive ways such as how children are moving their bodies. Another alternative is to set pre-determined timers to take short breaks.

### 3.3. Social Interactions in Virtual Reality

Many factors affect children’s and adolescents’ attraction to video games, one being their ability to share the experience with friends [49]. The use of social interactions may be particularly effective during adolescence, a time in which children are particularly sensitive to social environments [50]. Children as young as five years old respond to digital others in social ways, such as using information from a virtual character and a live person at equal rates to solve a decision-making task [51].

Children with chronic pain may have particular deficiencies in their abilities to develop and maintain peer relationships due to increased school absences, decreased physical ability to engage in sports or social activities, and decreased mood and motivation [52]. Virtual environments may offer an alternative platform for children to build these relationships by giving them access to near-real experiences that they can share with others. Children with high medical acuity, such as those in post-transplant oncology, who may be confined to a single room or unit, may benefit from access to virtual interactions if they are not able to engage with peers due to their ongoing medical condition. Since older children are already actively involved in virtual environments [53], the presence of virtual others offers new opportunities. For example, physical therapists might incorporate their own behavior into the environment via an avatar, which could help patients visualize or mimic the correct movements.

However, as in real life, children need age-appropriate environments. Social virtual environments where children can interact with others such as High Fidelity (highfidelity.io) or AltspaceVR (altVR.com) may require additional permissions so that outsiders cannot interact with patients. In addition, the risks and benefits of social media are also applicable when social media platforms provide opportunities for patients to engage virtually. While Facebook’s current beta application, Spaces (oculus.com/experiences/rift/), and other environments linked with social media platforms may make it easy for children to engage with Facebook friends in immersive virtual reality, such interactions should not allow children to isolate themselves from face-to-face interactions.

### 3.4. Customization

Clinicians or patients themselves can select different virtual scenarios, and as commercial VR experiences become more common, there will be more entertainment options from which to choose. Allowing children to contribute towards selecting their virtual experience allows them agency and the ability to tailor content to their interests, as in a study by Ni et al. [54] in which pediatric patients and therapists evaluated games for degree of engagement and therapeutic usefulness together. For example, children undergoing chemotherapy who selected from a variety of virtual scenarios demonstrated reduced symptom distress [55].

Another area in which customization can be advantageous is in avatar representation. Children can select and customize their own avatar, providing them with a sense of control in a clinical setting that can significantly reduce patient stress [56]. When embodying stock avatars, children may need guidance in selecting age-appropriate material, as research suggests that users may conform their behavior to their preconceptions about the avatars they embody [57,58].

### 3.5. Embodiment

When body trackers, such as hand trackers, are used in virtual reality, participants can move their tracked limbs in real life and see their movements represented by the movements of their avatars in virtual reality. Thus, when patients are embodied in an avatar, they gain the sense that their avatar body has replaced their real body. This allows interventions that are possible in no other medium.

The tracking capabilities inherent in embodied VR experiences also offer clinicians the ability to monitor their patients’ physical movements and quantify rehabilitative effort without relying on self-report data. As wearable monitors for tracking physiological data such as gait and posture become increasingly used, clinicians may observe their patients on multiple levels and tailor their treatment accordingly. As with all patient data, movement data in children must be carefully protected.

Movement is not the only aspect of patient activity that can be visualized. Biofeedback is one arena that would lend itself well to the flexibility of virtual reality, allowing the visualization of pain-linked physiological signals. This could be particularly useful for children who may not be as adept as adults at verbally describing sensory information, or translating verbal instruction into action. An example is described in a recent pilot study mentioned above, where non-immersive VR mirror visual feedback was used in combination with biofeedback to treat pediatric patients with chronic headaches [56].

In VR, patients can also be embodied in novel avatars, or avatars that do not conform to the limitations of their physical bodies [57,58]. As discussed above, Jäncke et al. propose that children may be more susceptible than adults to developing a feeling of presence in virtual environments, even in novel avatar bodies [59]. Thus, as with virtual environments, the experience of embodiment in an avatar, especially an avatar that is dissimilar to a child’s real body must be contextualized. Debriefing post-VR may be necessary.

## 4. Practical Aspects of Virtual Reality: Hardware and Software

Clinicians looking to integrate virtual reality into their practice may benefit from the following information about the strengths and limitations of current consumer VR set-ups.

At a minimum, virtual reality requires a display in which the user sees the virtual environment. In the most common consumer systems, this is done through a head-mounted display (HMD). An HMD is a type of VR headset that displays digital images on two screens in front of the user’s eyes. In phone-based VR systems such as Gear VR, Google Cardboard or Google Daydream, the HMD consists of a smartphone wrapped in an inexpensive case with lenses, such that the phone provides both the computing power and display. In more powerful VR systems, such as the Oculus Rift or HTC Vive, the content displayed on the headset is generated by a desktop or laptop computer certified as “VR ready” by the manufacturer, or, in the case of PlayStation, by a video game console. In all of these systems, the user’s movements are tracked, and the system updates the content that is displayed to the user based on these tracked movements. Thus, as a child turns her head when looking around a virtual environment through an HMD, the content updates on the screens in front of her eyes, just as it would in the physical world if she turned her head to look at a different part of the room. Brief descriptions of some currently available consumer systems are found in Table 3.

### 4.1. Tracking Movement

**Figure 1:**
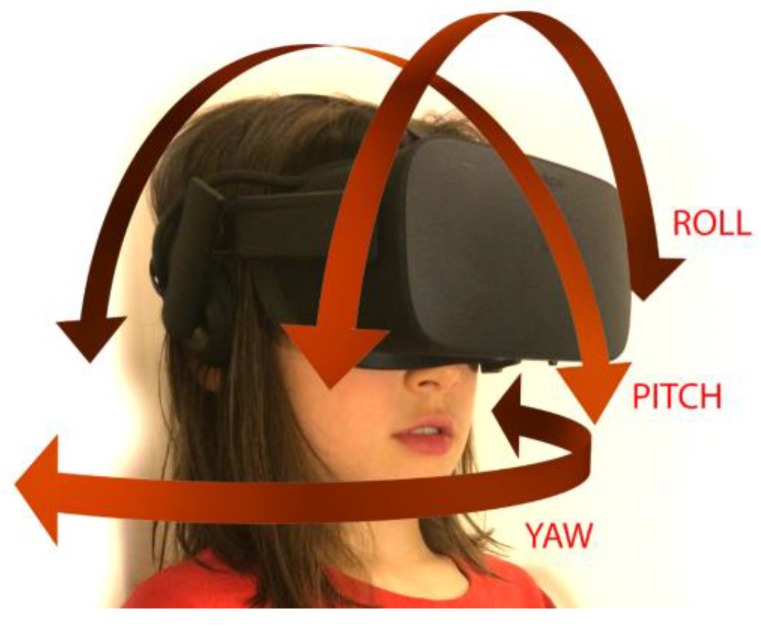
The child may move her head in pitch orientation, as in nodding her head, in yaw orientation, as in moving her head from side to side to look around the environment, or in roll orientation, as in touching her ear to her shoulder.

Tracking users’ movements while they experience VR is a key factor in creating realistic and compelling scenarios [1]. Two kinds of tracking are used in VR: orientation and position. Orientation, shown in Figure 1, tracks the user’s head movements and allows her to gaze around her virtual environment.

In VR systems that only have orientation, users are unable to move through scenes using their own body movement (i.e., walking). Instead, users might navigate through virtual environments using gaze, touchpad input or other less-embodied navigation techniques. Currently, smartphone-based devices tend to track only head orientation. While these systems are more limited in some respects, they are generally portable and self-contained, and require very little set-up time or space to use. They are also lighter, cheaper and potentially easier to clean and/or have disposable components, all of which may provide significant advantages when using them for pediatric patients.

**Figure 2:**
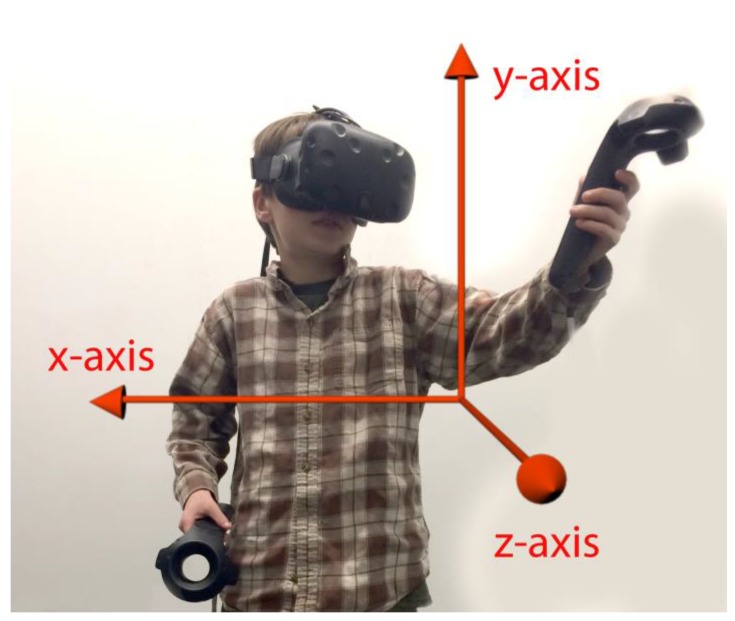
Virtual Reality (VR) coordinate system. In this picture, movement in the *y*-axis corresponds to moving up and down, movement in the *x*-axis corresponds to moving left and right, and movement in the *z*-axis corresponds to moving forward and backward. While global positional coordinates may vary according to set up, *y* is the up–down direction and the *z*-axis will often reflect movement towards the monitor of the desktop computer.

In addition to orientation tracking, systems can also have positional tracking (e.g., moving forwards or backwards in VR). Position, shown in Figure 2, tracks the user’s body and allows him to relocate within his environment. If the user holds hand trackers, his or her hands will also be tracked. This allows the user to interact with objects in the environment using his or her hands, and optionally see his or her hands represented by an avatar’s hands. Positional tracking typically uses external sensors placed around a room that capture the position of the headset and/or additional tracked objects. Such systems allow users to walk around in the virtual space.

While they increase the level of interaction available, systems with sophisticated tracking capabilities that include hand trackers tend to be more expensive than those that only capture orientation. While still portable to an extent, more complex systems also require sufficient space for patients to safely move around, and the headsets may be heavier. They will also require more set-up time.

### 4.2. Hardware Issues to Consider for Children

Although consumer headsets are designed to be adjustable, they can be too heavy or too large to be easily used by smaller children, as Dahlquist, et al. speculate in their 2009 study [41]. An additional issue for hospital use is that headsets, along with hand-tracking devices, which come into close contact with users’ bodies, will need to be cleaned.

These issues and more can be easily addressed through modular additions and adaptations to the basic VR unit, which would allow the technology to be effective in a wide range of clinical situations. Some consumer systems have washable covers to protect the parts of the headset that come into contact with patients’ skin. Cleansing wipes can also be used on both trackers and sensors. In a 2014 study on a patient who had burn wounds on his head, a consumer HMD was mounted to an articulated arm so that the patient did not actually need to wear the device [9]. Very young children may also need the unfamiliar equipment to be decorated or disguised to be more appealing. As one of the authors has found, by introducing an HMD as a mask with a soft cover designed with a friendly face, children were more open to wearing the virtual reality equipment [60]. If trends in lightness, portability, and decreased price continue, VR systems are likely to become increasingly better adapted for pediatric patients.

### 4.3. Virtual Content

Researchers seeking virtual content have three options. They may use existing free or inexpensive consumer content. They may develop their own content. Finally, they may work with industry partners focused on the clinical use of virtual reality. The following section lists some resources available at press time.

Free or pre-existing games designed for virtual reality are becoming increasingly available and may be an appropriate option, particularly for procedural pain. Such games can be purchased and downloaded; for example, through the Steam and Oculus libraries. Another type of frequently free virtual content is 360° videos, which can be downloaded directly to the device. Some main content libraries are currently YouTube360, Within, Jaunt, NYTVR, Condition One, WSJ, and LifeVR [61]. Most content in these libraries consists of 360° videos, which allow for orientation tracking without much interaction. Finally, social interactions in a virtual space can be facilitated using social platforms such as Facebook Spaces, High Fidelity, AltSpaceVR, etc.

Virtual content can be also created for specific clinical purposes using development platforms such as Unity3D (unity3d.com), Unreal (unreal.com) and Vizard (vizard.com). Researchers seeking to create customized content, which might include those investigating rehabilitation or neurorehabilitation, may require specialized tracking and rendering or customization. Such researchers might consider collaborating with university departments, for example computer science, information science, or communication departments, or work with existing software companies. Recording one’s own immersive video is also an option. Cameras ranging in complexity and price from the hundreds to thousands of dollars allow researchers to record their own video content and prepare it for viewing through a phone-based HMD.

Finally, there is increasing commercial interest in supplying clinicians with virtual reality applications. While it is outside the scope of this paper to recommend specific companies, searching for “virtual reality therapy” online will provide a list of suppliers. In addition, it is possible to make some recommendations for clinicians to take into consideration when working with a supplier. If patient data is collected as part of treatment, it may raise confidentiality issues. Developers should have experience working in a clinical setting. Compatibility with hardware should also be considered, as some application developers use proprietary hardware, while others allow the use of consumer systems. A brief list of currently available free games can be found in Table 4, below.

While more research is necessary to determine the more complex aspects of VR in pediatric care, VR can be used immediately to improve the quality of life for pediatric patients. A clinician may order one of the above-mentioned devices to be kept in the clinic and made available to patients who require one of the procedures described earlier in this review. For example, a child might be given a headset and immersed in a relaxing forest scene while they are receiving an immunization or IV placement. Alternatively, a child requiring frequent dressing changes, or other painful procedures may choose to purchase a unit for home use to assist with pain management. A patient with chronic pain may find it beneficial to use VR interventions to temporarily alleviate symptoms, or to assist in performing challenging tasks while in physical or occupational therapy. The number of applications is unlimited, and can be adapted to the individual child’s interests and the clinical therapeutic need.

## 5. Conclusions

In summary, VR is a promising new technology that offers unique opportunities to modulate the experience of pain. These opportunities include the management of acute and procedural pain and familiarizing children with future procedures via simulation. In addition, extrapolating from current evidence in adults, we propose that virtual reality may assist in the treatment of pediatric chronic pain via neuromodulation, as well as physical therapy.

Given the reduction in cost and increased ease of access, we hope that clinicians will be encouraged to explore the potential of this new modality. While the immediate adoption of VR can already engage and entertain children in a clinical setting with potential therapeutic benefits, continued study of the applications and efficacy of virtual reality in the treatment of pediatric pain is needed to better understand the impact upon quality of life for pediatric patients.

## Figures and Tables

**Table 1:** The following terms were searched on PubMed (www.ncbi.nlm.nih.gov/pubmed/) between 2000 and 2017.

Key Word Search	Number of Articles Obtained	Number of Articles Deemed Relevant and Utilized in Review
Virtual Reality and Pediatric Procedures	94	13
Virtual Reality and Pediatric Anxiety	14	7
Virtual Reality and Procedural Anxiety	13	8
Virtual Reality and Pediatric Procedural Anxiety	5	5
Virtual Reality and Pediatric Chronic Pain	4	4
Virtual Reality and Pediatric Acute Pain	5	5
Virtual Reality and Pain	312	31
Virtual Reality and Acute pain	35	16
Virtual Reality and Chronic Pain	63	27

**Table 2:** Possible benefits and side effects of VR.

**Benefits**	Provides distraction from pain Promotes movement Promotes imagination Fosters sense of internal health locus of control Promotes cortical repatterning (potentially)
**Side Effects**	Visually-induced motion sickness (dizziness, nausea) Collisions with nearby objects As with other media, risks social isolation In younger children, possible potential for “false memories”

**Table 3:** Virtual Reality (VR) head-mounted display (HMD) hardware. This table provides a non-exhaustive list of hardware

Product Name	Pricing for Headset at Time of Publication	Product Information	Description	Appropriate Ages	Limitations	Tracking
**Head and Hand Tracking**						
HTC Vive	$799	https://www.vive.com/us/	HMD & hand trackers, whole-room VR	(minimum 7+)	Requires “VR ready” personal computer (PC)	Positional and rotational
Oculus Rift &Touch Controllers	$599.98	https://www.oculus.com/rift/	HMD & hand trackers, can be set up on a desktop	13+	Requires “VR ready” PC	Positional and rotational
PlayStation VR	$499	https://www.playstation.com/en-us/explore/playstation-vr/	Video game console HMD and hand trackers	12+	Requires Sony PS4, compatible only with PlayStation games	Positional and rotational
**Head Tracking**						
Google Cardboard	$5 and up	https://vr.google.com/cardboard/	Phone-based	Unspecified; with adult supervision (single use <5–10 min)	No hand tracking, limited interactivity; requires VR-compatible phone	Rotational
Google Daydream	$79	https://vr.google.com/daydream/	Phone-based; lightweight; includes controller	13+	Currently limited software library; requires VR-compatible phone	Rotational with one controller
Gear VR	$129.99	https://www.oculus.com/gear-vr/	Phone-based, adjustable headset with hand controller	13+	Limited features compared to PC-based VR; requires VR-compatible phone	Rotational

**Table 4:** Suggestions for VR games and their applications.

Game Title	Hardware Compatibility	Where to Find It	Potential Applications	Qualities
Google Earth VR	-HTC Vive -Oculus Rift	https://vr.google.com/earth/	-Anxiety -Distraction therapy -Procedural pain	-Hands-free -Cinematic -Engaging
Minecraft	-HTC Vive -Oculus Rift -Samsung -GearVR -Google -Cardboard	1. Install PC version of Minecraft 2. Install Vivecraft (http://www.vivecraft.org/) for VR compatibility	-Anxiety -Distraction therapy	-Controller required -Well-known by kids -Engaging
Guided Meditation VR	-HTC Vive -Oculus Rift -Samsung -GearVR	https://guidedmeditationvr.com/download/	-Anxiety -Distraction therapy -Procedural pain	-Hands Free -Calming
The Lab	-HTC Vive -Oculus Rift	http://store.steampowered.com/app/450390/The_Lab/	-Anxiety -Distraction therapy	-Exploration -Specific movements (archery + slingshot) -Scenic
The Blu	-HTC Vive -Oculus Rift	http://store.steampowered.com/app/451520/theBlu/	-Anxiety -Distraction therapy	-Hands-free -Cinematic -Exploration
